# Sleep Deprivation Influences Trial-to-Trial Transfer but Not Task Performance

**DOI:** 10.3390/jcm11195513

**Published:** 2022-09-20

**Authors:** Bingyao Shen, Zhiqiang Tian, Jiajia Li, Yu Sun, Yi Xiao, Rixin Tang

**Affiliations:** 1 Department of Psychology, School of Social and Behavioral Sciences, Nanjing University, Nanjing 210023, China; 2 National Key Laboratory of Human Factors Engineering, China Astronaut Research and Training Center, Beijing 100094, China; 3 School of Psychological and Cognitive Sciences, McGovern Institute for Brain Research, Peking University, Beijing 100871, China

**Keywords:** sleep deprivation, Fitts’ Law, trial-to-trial transfer, visuomotor memory, task performance, goal-oriented movement

## Abstract

Previous research has shown that sleep deprivation can affect emotions and some cognitive functions. However, research on how sleep deprivation influences the visuomotor memory have rarely been reported. In the current study, a Fitts’ Law task was used to investigate how movement and the visuomotor memory are affected under the condition of sleep deprivation. Experiment 1 had 36 participants (15 males, mean age = 21.61 years) complete the same Fitts’ Law task 10 days apart under standard conditions. Experiment 2 had five participants (three males, mean age = 27.2 years) complete the task after 7 days of sleep deprivation, then complete it again after 10 days without sleep deprivation. Experiment 1 demonstrated the stability of the trial-to-trial effects. Experiment 2 showed that the previous trial (n) exerted no effect on the current trial (n + 1) under the conditions of sleep deprivation (*p* = 0.672). However, the effect was observed after 10 days without sleep deprivation (*p* = 0.013). This suggests that sleep deprivation did not affect task performance but influenced the transfer of the trial history. Future studies are required to investigate the effect of sleep deprivation with more participants.

## 1. Introduction

Some professionals (e.g., pilots and medical staff) must live and work on duty by turns and potentially perform functions while coping with sleep deprivation. Astronauts, for instance, may need to manually operate complex technical systems with touch screens or monitors, requiring significant cognitive and psychomotor functions, while sleep deprived [1]. Even in daily life, people sometimes have to stay up late to complete their heavy workload before the deadline under conditions of sleep deprivation. In the meantime, more than 50% of the general population was affected by obstructive sleep apnea, which is a frequent respiratory sleep disorder and is associated with neurocognitive performance impairment [2]. Previous studies have mostly focused on the effect of sleep deprivation on emotions and some cognitive functions [3,4]. By contrast, research on how sleep deprivation affects hand movement and task performance, particularly after extended periods of sleep deprivation, have rarely been reported. Further, very few studies have investigated the impact on performance using systems involving touch screens or monitors, commonplace in space capsules, submarines, medical fields, and many other work contexts in which manual speed and accuracy are paramount.

Many studies have reported that sleep deprivation can adversely influence the emotional state [5]. For instance, research by Zohar et al. has shown that sleep deprivation can lead to an increase in negative emotion and a decrease in positive emotion [6]. In addition to these emotional issues, scientific observations and anecdotal reports during human spaceflight have provided evidence for cognitive dysfunction, including spatial disorientation, visual illusion, time sense disorder, impairment of attention and concentration, and disturbances of motor skills (for review, see [7]). Previous research has suggested that high emotional arousal facilitates the smoothness of movement [8] and several cognitive functions, such as attentional focus [9,10] and working memory [11], thus affecting motor learning. Daviaux et al. have found that sleep deprivation affects the perception of action capabilities via the cognitive processing of inputs [12]. However, there are also studies that did not show this effect. For example, Hennecke et al. have found that restricted sleep only impaired the spatial working memory but not the declarative memory [13]. Previous studies of three consecutive 12 h nursing shifts have shown that no significant differences were found in the mean reaction time between different shifts although neurobehavioral tests demonstrated performance problems after working for 12 consecutive hours [14]. Many previous results have also indicated that sleep deprivation does not inevitably influence performance on executive control components of cognition [15,16]. Meanwhile, it should be noted that professionals (e.g., astronauts and medical staff) working with sleep deprivation can still finish tasks correctly and perform their jobs successfully. Conflicting results could be accounted for by differences in the experimental paradigms and different durations of sleep deprivation. The cognitive impairments were affected differently by chronic sleep deprivation and acute sleep deprivation [17]. Previous research mostly focused on task performance but studies on cognition related to the performance have rarely been reported. The current study aimed to investigate the effects of 7 days of sleep deprivation on simple motor skills. At the same time, we investigated whether the effects on cognitive functions were in accordance with those on task performance. It is possible for hand movement to be affected by sleep deprivation directly or via emotions.

Fitts’ Law can be used to investigate the effects of sleep deprivation on precise hand movement. Fitts’ Law states that a stable functional relationship exists among distance, target size, and movement time in target-oriented movement and is suitable for a wide range of tasks, including the movements of the hand, handheld tools [18], or a mouse-controlled cursor [19]. Fitts’ Law describes the tradeoff between accuracy and time in an aiming task, which reflects both the task performance and visuomotor memory. The law has been widely studied in psychology and is suitable for a wide range of purposes, including investigating the impact of sleep deprivation on precise hand movements.

Previous Fitts’ Law studies have shown that the grasping movement in a trial is influenced by its occurrence in a previous trial [20]. It also occurs during a cursor-hitting task. Specifically, the movement time required to hit the current target manually or with a mouse is shortened when the target in the previous trial is large, and the movement time required to hit the current target is extended when the target in the previous trial is small [21]. Trial-to-trial transfer is explained as a transfer of a sensorimotor memory, indicating that the previous visuomotor memory plays an important role in programming the grasping movement [22]. However, the trial-to-trial transfer effect lasts for a short time and typically disappears by 5 s [23]. Based on these reports of our lab, we planned to study whether the trial-to-trial transfer would disappear under conditions of sleep deprivation if the visuomotor memory were damaged by sleep deprivation. We predict that trial-to-trial transfer is likely to disappear because of cognitive function impairment.

In the current study, a Fitts’ Law task was used to investigate whether a task performance or a transfer of the trial history was influenced by 7 days of sleep deprivation. It might be the case that sleep deprivation would affect the trial-to-trial transfer but not the task performance. Two experiments were conducted to investigate whether the movement performance and the transfer of the trial history were affected by sleep deprivation. In Experiment 1 (Exp1), the participants moved a cursor to hit the target under normal conditions when the target sizes were presented either blocked or alternately. In Experiment 2 (Exp2), the same was conducted on the participants after 7 days under conditions of sleep deprivation before they left the confined space. Participants were allowed 8 h of sleep every 24 h over a 7-day testing period. The participants in both Exp1 and Exp2 were tested 10 days later to provide baseline data. We predicted that the task performance was not affected by sleep deprivation, indicating that the cursor-hitting movement was a highly automated muscle movement with low sensorimotor complexity. However, the trial-to-trial transfer was expected to be influenced by sleep deprivation, indicating that the formation of the trial history needed high-level cognitive processes, which could be damaged by sleep deprivation.

## 2. Experiments 1A and 1B

### 2.1. Participants

Experiment 1A (Exp1A) included 42 participants (21 males, *M*_age_ = 22.13 years, *SD* = 2.46). All participants were right-handed and had normal or corrected-to- normal vision. Prior to the experiment, they provided informed consent, which was approved by the Ethical Committee of the Department of Psychology, Nanjing University. After at least 10 days, the same participants were asked to complete the cursor-hitting task. Thirty-six participants were left to undergo Experiment 1B (15 males, *M*_age_ = 21.61 years, *SD* = 2.32).

### 2.2. Apparatus and Procedure

Materials. The Positive and Negative Affect Schedule (PANAS) was used to assess the emotional state of the participants prior to each round [24]. The scale consisted of 20 words describing emotions. The participants were asked to read each word and then rate the intensity of elicited emotion on a 5-point scale (1 = not at all, 5 = very consistent).

**Figure 1:**
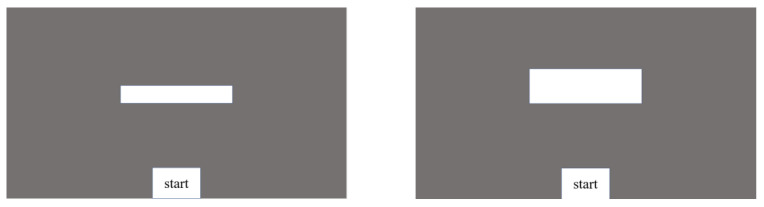
The target used in Experiment 1 was either a small rectangle or a large rectangle. The displays were presented either blocked or alternately. Thus, 4 kinds of movement from the current trial to the next trial were identified: large-to-large (L:L), small-to-small (S:S), small-to-large (S:L), and large-to-small (L:S).

Apparatus. Participants used a 13.3-inch laptop (Samsung 910S3L, 29.6 cm × 16.6 cm) with the resolution set to 1024 × 768.The viewing distance was set to approximately 50 cm. A white rectangular target, either small (8.6 cm × 1.1 cm) or large (8.6 cm × 2.3 cm), was presented in the gray background. At the bottom of the screen was a white start box (3 cm × 4 cm). The layout is presented in Figure 1.

Procedures and design. Each trial began with the presentation of a start box. Participants moved the mouse to hit the start box with a cursor until a target was presented. They were then asked to hit the white rectangular target as soon as it appeared and as precisely as possible. They moved the cursor back toward the start box and waited for the next target. Movement time (MT) was defined as the time that the participants moved the cursor from the start box to the target. The intertrial time interval was 3 s between the presentation of the target in the current trial to the presentation of the target in the next trial. The participants were allowed 10 to 12 practice trials to ensure that they knew how to perform the task. If the cursor fell outside the target more than twice, the trial was marked as an error, and the participants had to perform the task again.

**Figure 2:**
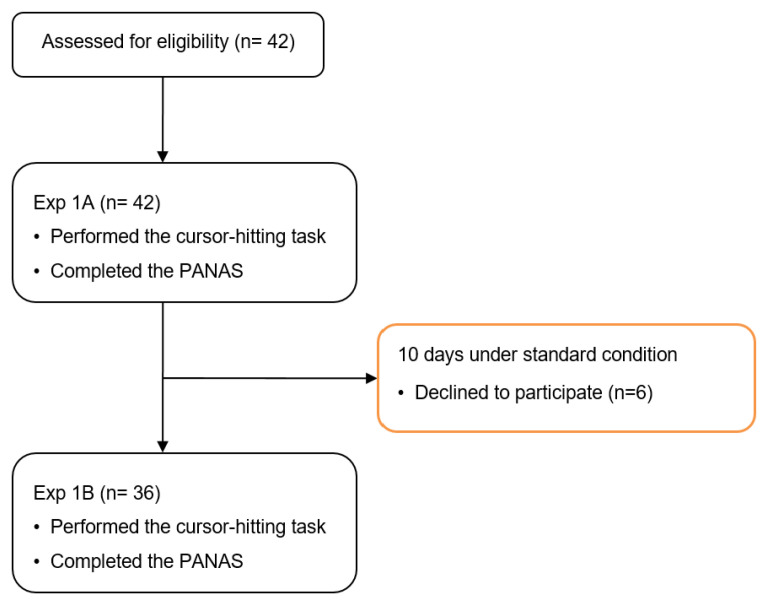
Consort flow diagram of Experiment 1. PANAS, The Positive and Negative Affect Schedule.

As shown in Figure 1, large and small targets were presented either blocked or alternately. Thus, four kinds of movements from the current trial to the next trial were identified: large-to-large (L:L), small-to-small (S:S), small-to-large (S:L), and large-to-small (L:S). In the blocked presentation, the small and large targets were displayed separately in 12 trials, whereas in the alternating presentation, the large and small targets were displayed alternately in 24 trials. Thus, 48 trials (12 × 2 (the current size: large or small) × 2 (the previous size: large or small) = 48) were performed under each condition, presented in a balanced order. The participants were allowed to rest for 60 s after each condition. Participants were required to complete a full round of trials; then, 10 days later, they completed a second round. PANAS was used to measure the intensity of emotion of the participants prior to Exp1A and Exp1B. The procedure is presented in Figure 2.

### 2.3. Results

**Figure 3:**
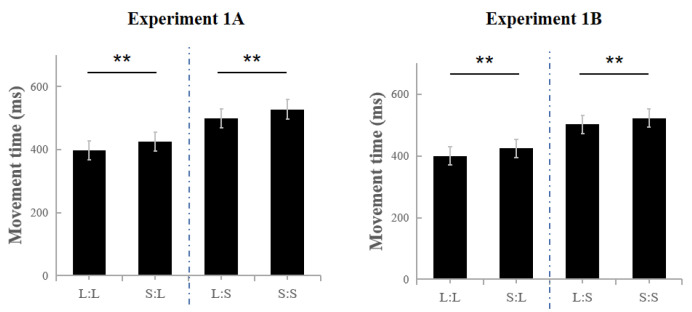
Results of Experiment 1A and 1B. In Exp1A, the movement time of L:L is significantly shorter than that of S:L, and the movement time of L:S is significantly shorter than that of S:S. Similar observations in Exp1B are noted. ** *p* < 0.01.

The day vs. 10 days later under the standard condition (Figure 3). A 2 (time: the first measurement vs. the second measurement) × 2 (previous size: large vs. small) × 2 (current size: large vs. small) repeated measures ANOVA was conducted using MT. The main effect of the current size was significant, F(1,35) = 836.078, *p* < 0.01, and *η*_p_^2^ = 0.960, and the MT of hitting the small target was longer than that of hitting the larger target, which conformed to Fitts’ Law. The main effect of the previous size was also significant, F(1,35) = 49.917, *p* < 0.01, and *η*_p_^2^ = 0.588, suggesting that hitting the large target in the previous trial shortened the current MT relative to that entailed by hitting the small target previously in both Exp1A and Exp1B. These results indicate that a trial-to-trial transfer occurred. The main effect of time was not significant (F(1,35) = 0.018, *p* = 0.894, and *η*_p_^2^ = 0.001), which showed that neither the practice nor the time exerted an effect on the performance. Similarly, other interactions showed no significance (F(1,35)_max_ = 0.298, *p*_min_ = 0.589, and *η*_p_^2^ = 0.008).

Changes in emotion under the standard condition. The positive affect (M ± SD: Exp1A: 25.75 ± 7.62; Exp1B: 25.11 ± 7.17) and negative affect under a standard condition and 10 days later were assessed (M ± SD: Exp1A: 13.44 ± 4.42; Exp1B: 14.47 ± 6.31). The paired *t*-test showed no significant difference between the first measurement and the second measurement (positive affect between the 1st and 2nd trials: t = 0.366, *p* = 0.716; negative affect between the 1st and 2nd trials: t = 0.730, *p* = 0.471).

## 3. Experiments 2A and 2B

Exp1A and Exp1B verified the existence of the trial history under the standard condition and showed that the task performance was not affected by the practice trials. Exp2A and Exp2B were conducted to determine whether the trial-to-trial transfer and task performance (i.e., movement time) were influenced under the conditions of sleep deprivation for an extended time.

### 3.1. Participants

Six adults were initially recruited in Experiment 2 but only 5 completed all of the data collection. Five adults (3 males, *M*_age_ = 27.2 years, *SD* = 1.94) participated in Exp2A and Exp2B. All participants were right-handed and had normal or corrected-to-normal vision. The participants stayed in a closed room with sleep deprivation for 7 days. Written informed consent was obtained from all participants, and the experiment was conducted in accordance with the guidelines approved by the Ethics Committee of China Institute of Marine Technology and Economy.

### 3.2. Apparatus and Procedure

Experimental stimuli were presented on a 14-inch laptop (ASUS laptop, 31.2 cm × 17.5 cm) in Exp2. Other settings, such as the experimental materials, resolution, and cursor-hitting task, were identical to those in Experiment 1. PANAS was used to measure the emotional state of the participants 1 d before the participants were placed under the condition of sleep deprivation and on the last day of sleep deprivation. Exp2A and Exp2B used the same procedure as Exp1, except that the participants in Exp2A were initially instructed to stay in a confined room with sleep deprivation for 7 days. The same participants were recruited again to participate in Exp2B after 10 days without sleep deprivation.

**Figure 4:**
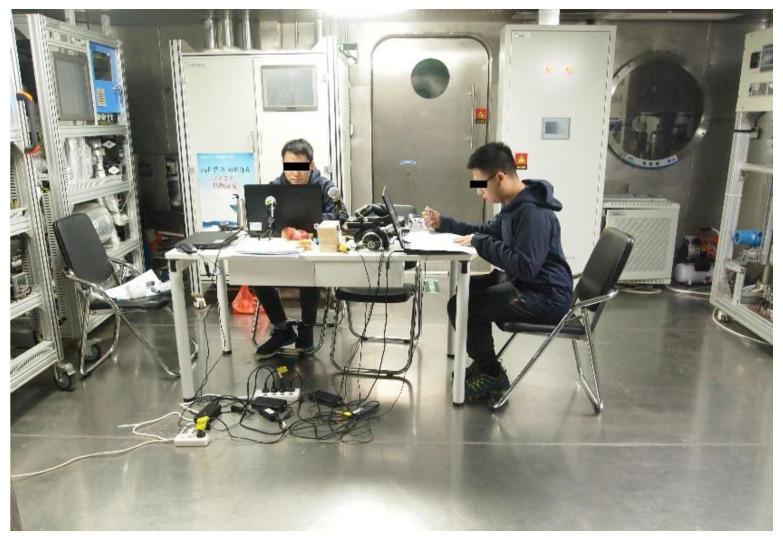
Scenario of sleep deprivation.

**Figure 5:**
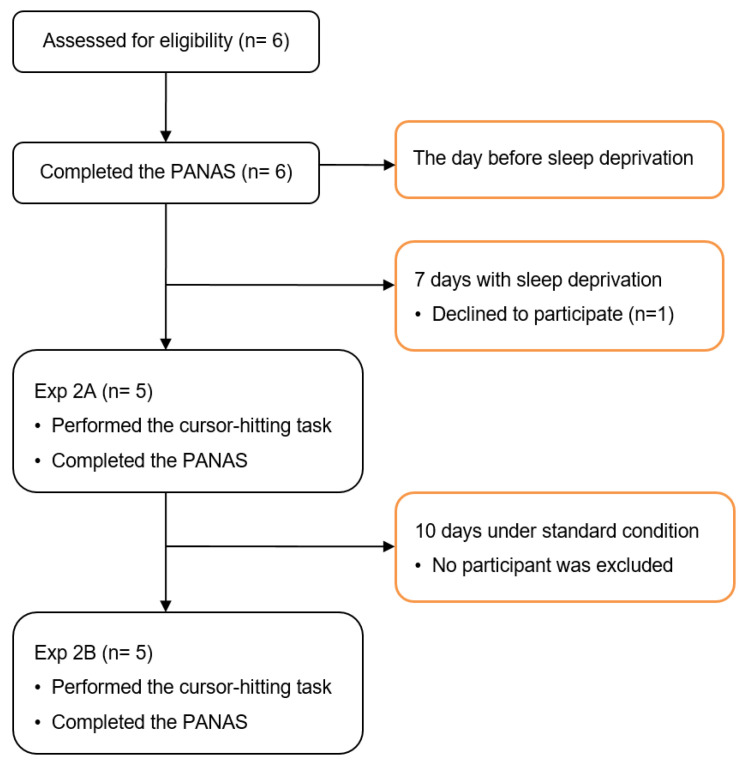
Consort flow diagram of Experiment 2. PANAS, The Positive and Negative Affect Schedule.

Participants maintained sleeping and waking periods with normal 8 h of sleep for three nights prior to the experiment. To exclude the influence of different daily activities, the sleep deprivation experiment was conducted in a confined space, with each participant having a separate resting area and a public activity area (Figure 4). The participants were allowed to carry only paper books and materials when they entered the confined space. The space was equipped with a good ventilation system, and adequate food and water were supplied regularly. The room was illuminated with less natural light. The participants were instructed to perform their normal daily activities with 24 h sleep deprivation, following an 8 h sleep pattern. Then, the participants suffered a new 24 h sleep deprivation period, and the next sleep–awake cycle started. The participants’ sleep–wake cycles were supervised. The procedure is presented in Figure 5.

### 3.3. Results

**Figure 6:**
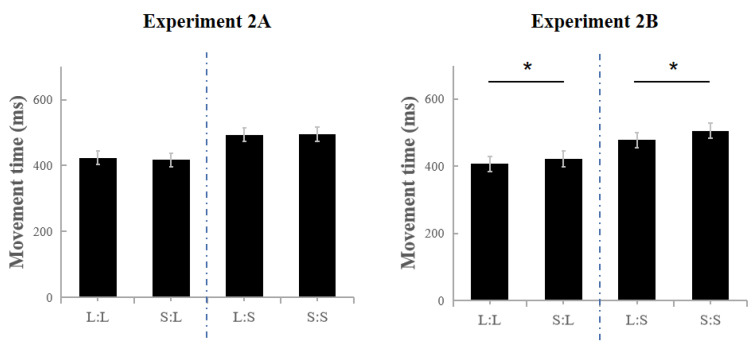
Results of Experiment 2A (Exp2A) and Experiment 2B (Exp2B). In Exp2A, no significant difference in movement time (MT) is found between L:L and S:L; moreover, the MT of L:S is similar to that of S:S. However, in Exp2B, the MT of L:L is as marginally significant as that of S:L, and the MT of L:S is significantly shorter than that of S:S. * *p* < 0.05.

Under conditions of sleep deprivation vs. after 10 days without sleep deprivation (Figure 6). A repeated measures ANOVA on the MT was first carried out for conditions of sleep deprivation and after 10 days without sleep deprivation. A 2 (Condition: Sleep deprivation vs. Without sleep deprivation) × 2 (Previous size: large vs. small) × 2 (Current size: large vs. small) repeated measures ANOVA was conducted for the MT. The results indicate that the main effect of the current size was significant, F(1,4) = 117.452, *p* < 0.01, and *η*_p_^2^ = 0.967, and the MT for hitting a small target was longer than that for hitting a large target, which conforms to Fitts’ Law. The interaction between the condition and previous size was significant, F(1,4) = 17.588, *p* = 0.014, and *η*_p_^2^ = 0.815. The results of the simple effect analysis show that the previous size affects the current MT after 10 days without sleep deprivation (*p* = 0.013) but not while under conditions of sleep deprivation (*p* = 0.672). Thus, the sleep deprivation affected the trial-to-trial transfer. No significant effect was found in the two-way interaction between the condition and current size, F(1,4) = 0.069, *p* = 0.806, and *η*_p_^2^ = 0.017. This finding suggests that sleep deprivation exerted no effect on task performance. No other significant main effects and interactions were determined—that is, (F(1,4)_max_ = 3.181, *p*_min_ = 0.149, and *η*_p_^2^ = 0.443).

**Figure 7:**
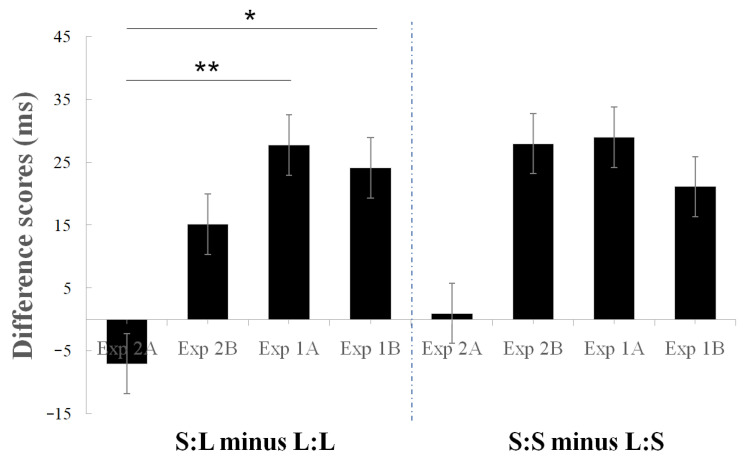
Difference scores in Experiment 1 and Experiment 2. The difference scores in S:L and L:L are significantly different between Experiment 2A (Exp2A) and Experiment 1A (Exp1A) and between Exp2A and Experiment 1B. * *p* < 0.05, ** *p* < 0.01.

Difference scores. The difference scores between the blocked and alternating conditions were calculated to rule out some irrelevant effects and focus on the trial-to-trial effect. The difference scores (S:L minus L:L and S:S minus L:S) of Exp2A (confined space) and the difference scores of the three other experiments (Exp1A, Exp1B, and Exp2B) were compared using paired *t*-tests or Welch’s *t*-test (Figure 7). Welch’s *t*-test is more reliable when the two samples have unequal variances or sample sizes. It also provides improved control of Type I error rates [25,26], rendering it suitable for a comparison among Exp2A, Exp1A, and Exp1B. Welch’s *t*-test illustrated that the difference scores between S:L and L:L were significantly different in the comparison between Exp2A and Exp1A (t(13.7) = 3.391, *p* = 0.005) and between Exp2A and Exp1B (t(15.1) = 2.907, *p* = 0.011). No other significant difference was found (t(4)_max_ = 1.909, *p*_min_ = 0.129).

Changes in emotion with sleep deprivation. The positive affect (M ± SD: prior to Exp2A: 28.90 ± 11.23; Exp2A: 24.13 ± 6.62) and negative affect of the participants (M ± SD: prior to Exp2A: 12.10 ± 1.24; Exp2A: 10.20 ± 0.45) the day before they started sleep deprivation and on their last day in the confined space were noted. The paired *t*-test indicated a significant difference in the negative affect between the day before the participants suffered from sleep deprivation and the last day they were in the confined space (t = 3.167, *p* = 0.034) but not in the positive affect (t = 1.504, *p* = 0.207).

## 4. Discussion

In this study, the effect of sleep deprivation on movement (particularly, trial history and movement performance) was investigated. The results of Exp1 demonstrate that trial-to-trial transfer occurred. The trial history was found to influence the current trial under the standard condition and after 10 days, which was consistent with previous studies in which the cursor-hitting task was conducted [21,23]. In Exp2, the previous trial exerted no effect on the current trial under the conditions of sleep deprivation. However, the effect was observed after 10 days without sleep deprivation. This suggests that sleep deprivation did not affect task performance but influenced the trial history.

Sleep deprivation affected trial-to-trial transfer in this study, which may partly be attributed to the interference with the visuomotor memory. Short- or long-term sleep deprivation was found to affect cognitive functions, including reaction time, working memory, and attention [27,28]. For instance, different types of sleep deprivation were reported to exert visual working memory impairment to different degrees [29]. Sleep deprivation was also found to affect the perception of action capabilities via the cognitive processing of inputs [12]. Evidence suggests that a lack of sleep is an important and common factor leading to fatigue for people who work shifts [30]. Fatigue can further lead to the decline of psychological, emotional, and other functions [31]. Therefore, although no significant difference in task performance was found, a change in the visuomotor memory was observed, suggesting that sleep deprivation negatively affected the visuomotor memory and motor learning.

The trial-to-trial transfer was also likely to be affected by sleep deprivation via emotions. Sleep deprivation can cause fluctuations in emotions [5]. In the current study, sleep deprivation indeed induced changes in the negative affect. The negative affect caused by sleep deprivation might directly relate to the trial-to-trial transfer. High emotional arousal was suggested to facilitate the smoothness of movement [8] and was favorable for an intimate relationship between emotions and the motor system [32,33]. Meanwhile, emotion was expected to influence the working memory [34,35], but the mechanism by which emotions affect the visuomotor memory has yet to be determined. Therefore, another possibility was that the negative affect destroyed the transfer by damaging the visuomotor memory. However, the current study is insufficient because the cursor-hitting task and emotion measurement were only performed on the day before the participants suffered from sleep deprivation and their last day with sleep deprivation. The continuous changes in participants under conditions of sleep deprivation over a period of 7 days could not be determined.

The current results find that sleep deprivation did not affect automated muscle skills but harmed the visuomotor memory, leading to the disappearance of the effect of the trial history. This was consistent with the processing efficiency model, which emphasizes that individuals initiate performance protection strategies under cognitive anxiety, thus concealing the influence of stress on task performance [36]. The compensatory response proposed by Frenda and Fenn also explains the current results, emphasizing that the brain can compensate for certain impairments brought on by sleep deprivation [37]. As a result, optimal performance may be maintained, which is consistent with our results. In addition, previous studies on soldiers have found that the reaction time and the accuracy of discrimination between friendly and enemy targets were significantly affected by 72 h of sleep deprivation despite stable shooting accuracy [38]. In contrast, some previous studies have shown that sleep deprivation exerted no effect on highly automated sensorimotor skills, such as handwriting kinematics and grip strength, but influenced handwriting performance in complex perceptual–motor tasks [39]. It is worth mentioning that handwriting kinematics is a highly automated sensorimotor skill, while hitting movements require real-time information for programming. The previous studies of our lab found that people would activate the corresponding motor programming based on the features of the objects [22]. One possible explanation for the conflicting results is that sleep deprivation impaired the ability to adjust according to the features of the objects and the trial-to-trial transfer.

The effect of the trial history was observed again after 10 days without sleep deprivation, indicating that cognitive impairment caused by sleep deprivation could be recovered. This finding was in accordance with previous research of Moore et al., showing that the cognitive reserve of astronauts in a sleep restriction cohort could recover to baseline after days [40]. The main limitation of the current study is that the period of 7 days for sleep deprivation might not be the shortest time for the absence of the trial-to-trial transfer. Whether or not the effect of the trial history would disappear after a shorter period of isolation remains a question for future experiments. For example, a follow-up study with different times of sleep deprivation would further explore how increasing the time of exposure to sleep deprivation influences the trial-to-trial transfer of the cursor-hitting movement. Additionally, the number of participants in Exp2 is low, which might have an influence on the statistical confidence and should be considered when conducting future experiments.

In summary, the current study investigated whether movement was affected by sleep deprivation, as well as determined the mechanism underlying this effect. The results show that sleep deprivation did not affect the task performance but influenced the trial history. Sleep deprivation ensured performance via a performance protection strategy but led to the decline in the visuomotor memory, as reflected in the disappearance of the effect of the trial history. Potential threats to human behavior under conditions of sleep deprivation exist due to the decline of cognition ability. The current study suggests that potential cognitive impairment must be considered under conditions of sleep deprivation even if the task performance is not influenced by sleep deprivation.

## References

[1] Kanas N., Manzey D. (2008). Space Psychology and Psychiatry.

[2] Pollicina I., Maniaci A., Lechien J.R., Iannella G., Vicini C., Cammaroto G., Cannavicci A., Magliulo G., Pace A., Cocuzza S. (2021). Neurocognitive performance improvement after obstructive sleep apnea treatment: State of the art. Behav. Sci..

[3] Killgore W.D. (2010). Effects of sleep deprivation on cognition. Prog. Brain Res..

[4] Tomaso C.C., Johnson A.B., Nelson T.D. (2020). The effect of sleep deprivation and restriction on mood, emotion, and emotion regulation: Three meta-analyses in one. Sleep.

[5] Pilcher J.J., Huffcutt A.I. (1996). Effects of Sleep Deprivation on Performance: A Meta-Analysis. Sleep.

[6] Zohar D., Tzischinsky O., Epstein R., Lavie P. (2005). The Effects of Sleep Loss on Medical Residents’ Emotional Reactions to Work Events: A Cognitive-Energy Model. Sleep.

[7] Christensen J.M., Talbot J.M. (1986). A review of the psychological aspects of space flight. Aviat. Space Environ. Med..

[8] Kang G.E., Gross M.M. (2016). The effect of emotion on movement smoothness during gait in healthy young adults. J. Biomech..

[9] Vaz D.V., Avelar B.S., Resende R.A. (2019). Effects of attentional focus on movement coordination complexity. Hum. Mov. Sci..

[10] Hilde K., Femke V.A., Sanne V., Sandra V.C., Bert S. (2018). Motor learning and movement automatization in typically developing children: The role of instructions with an external or internal focus of attention. Human Movement Science.

[11] Abswoude F.V., Buszard T., Kamp J.V.D., Steenbergen B. (2020). The role of working memory capacity in implicit and explicit sequence learning of children: Differentiating movement speed and accuracy. Hum. Mov. Sci..

[12] Daviaux Y., Mignardot J.-B., Cornu C., Deschamps T. (2014). Effects of total sleep deprivation on the perception of action capabilities. Exp. Brain Res..

[13] Hennecke E., Lange D., Steenbergen F., Fronczek-Poncelet J., Elmenhorst D., Bauer A., Aeschbach D., Elmenhorst E.-M. (2020). Adverse interaction effects of chronic and acute sleep deficits on spatial working memory but not on verbal working memory or declarative memory. J. Sleep Res..

[14] Geiger-Brown J., Rogers V., Trinkoff A.M., Kane R.L., Bausell R.B., Scharf S.M. (2012). Sleep, Sleepiness, Fatigue, and Performance of 12-Hour-Shift Nurses. Chrono-Int..

[15] Bratzke D., Steinborn M.B., Rolke B., Ulrich R. (2012). Effects of Sleep Loss and Circadian Rhythm on Executive Inhibitory Control in the Stroop and Simon Tasks. Chrono-Int..

[16] Fournier L.R., Hansen D.A., Stubblefield A.M., Van Dongen H.P.A. (2020). Action plan interrupted: Resolution of proactive interference while coordinating execution of multiple action plans during sleep deprivation. Psychol. Res..

[17] Dinges D.F., Pack F., Williams K., Gillen K.A., Powell J.W., Ott G.E., Aptowicz C., Pack A.I. (1997). Cumulative Sleepiness, Mood Disturbance, and Psychomotor Vigilance Performance Decrements During a Week of Sleep Restricted to 4–5 Hours per Night. Sleep.

[18] Fitts P.M. (1954). The information capacity of the human motor system in controlling the amplitude of movement. J. Exp. Psychol..

[19] Radwin R.G., Vanderheiden G.C., Lin M.-L. (1990). A Method for Evaluating Head-Controlled Computer Input Devices Using Fitts’ Law. Hum. Factors: J. Hum. Factors Ergon. Soc..

[20] Whitwell R.L., Lambert L.M., Goodale M.A. (2008). Grasping future events: Explicit knowledge of the availability of visual feedback fails to reliably influence prehension. Exp. Brain Res..

[21] Tang R., Shen B., Sang Z., Song A., Goodale M.A. (2018). Fitts’ Law is modulated by movement history. Psychon. Bull. Rev..

[22] Tang R., Whitwell R.L., Goodale M.A. (2015). The influence of visual feedback from the recent past on the programming of grip aperture is grasp-specific, shared between hands, and mediated by sensorimotor memory not task set. Cognition.

[23] Shen B., Liu Q., Song A., Wang X., Tang R. (2020). How long is the interval over which trial-to-trial effects on Fitts’ Law task can operate?. Exp. Brain Res..

[24] Watson D., Clark L.A., Tellegen A. (1988). Development and validation of brief measures of positive and negative affect: The PANAS scales. J. Pers. Soc. Psychol..

[25] Derrick B., Toher D., White P. (2016). Why Welch’s test is Type I error robust. Quant. Methods Psychol..

[26] Ruxton G.D. (2006). The unequal variance *t*-test is an underused alternative to Student’s *t*-test and the Mann–Whitney U test. Behav. Ecol..

[27] Feyer W. (2000). Moderate sleep deprivation produces impairments in cognitive and motor performance equivalent to legally prescribed levels of alcohol intoxication. Occup. Environ. Med..

[28] Ghanbari I., Taheri H.R., Sohrabi M. (2019). The Effects of 24-Hour Sleep Deprivation on Cognitive and Motor Skills of Male College Students. Ann. Appl. Sport Sci..

[29] Drummond S.P.A., Anderson D.E., Straus L.D., Vogel E.K., Perez V.B. (2012). The Effects of Two Types of Sleep Deprivation on Visual Working Memory Capacity and Filtering Efficiency. PLoS ONE.

[30] Dohrmann S.B., Leppin A. (2016). Determinants of seafarers’ fatigue: A systematic review and quality assessment. Int. Arch. Occup. Environ. Health.

[31] Allen P., Wadsworth E., Smith A. (2008). Seafarers’ fatigue: A review of the recent literature. Int. Marit. Health.

[32] Beatty G.F., Cranley N.M., Carnaby G., Janelle C.M. (2016). Emotions predictably modify response times in the initiation of human motor actions: A meta-analytic review. Emotion.

[33] Simoes Matos Saraiva A.C. (2017). Motion and Emotion: How Emotional Stimuli Influence the Motor System. Ph.D. Thesis.

[34] Schoofs D., Preuß D., Wolf O.T. (2008). Psychosocial stress induces working memory impairments in an n-back paradigm. Psychoneuroendocrinology.

[35] Luethi M., Meier B., Sandi C. (2009). Stress effects on working memory, explicit memory, and implicit memory for neutral and emotional stimuli in healthy men. Front. Behav. Neurosci..

[36] Eysenck M.W., Calvo M. (1992). Anxiety and Performance: The Processing Efficiency Theory. Cogn. Emot..

[37] Frenda S.J., Fenn K.M. (2016). Sleep less, think worse: The effect of sleep deprivation on working memory. J. Appl. Res. Mem. Cogn..

[38] Smith C.D., Cooper A.D., Merullo D.J., Cohen B.S., Heaton K.J., Claro P.J., Smith T. (2019). Sleep restriction and cognitive load affect performance on a simulated marksmanship task. J. Sleep Res..

[39] Jasper I., Häusler A., Baur B., Marquardt C., Hermsdörfer J. (2009). Circadian Variations in the Kinematics of Handwriting and Grip Strength. Chrono- Int..

[40] Moore S., Dilda V., Morris T., Yungher D., MacDougall H., Wood S. (2019). Long-duration spaceflight adversely affects post-landing operator proficiency (Version 1). Sci. Rep..

